# Functional and Patient-related Outcomes of Total Hip Arthroplasty in Patients Younger Than 20 Years

**DOI:** 10.1016/j.artd.2023.101100

**Published:** 2023-03-06

**Authors:** Antoine Chapot, Pierre-Yves Zambelli, Sophie Rosa Merckaert

**Affiliations:** a Centre Hospitalier Universitaire Vaudois, Service de chirurgie orthopédique pédiatrique, Lausanne, Switzerland; b Centre Hospitalier Universitaire Vaudois, Service d’Orthopédie adulte, Lausanne, Switzerland

**Keywords:** Total hip arthroplasty, Oxford hip score, Harris hip score, Paediatric hip disease, Avascular femoral head necrosis

## Abstract

**Background:**

Total hip arthroplasty (THA) in adolescent and young adults represent only about 10% of all THAs. Despite the advances in hip conservation surgery, there are still adolescents and young adults who progress to severe joint degeneration. THA seems to be the last solution in these cases. We aimed to assess the clinical and patient-related outcomes at short-term to midterm follow-up for THAs performed before the age of 20 years.

**Material and methods:**

We performed a retrospective monocentric study including all patients that underwent a THA before the age of 20 years between January 2008 and December 2018 at our tertiary orthopaedic center with a minimum follow-up of 2 years. Demographic data were recorded. The Harris and Oxford hip scores were used to assess clinical and patient-related outcomes.

**Results:**

A total of 11 patients (12 THAs) were included. Juvenile inflammatory arthritis and avascular necrosis due to slipped capital femoral epiphysis were the most commonly encountered etiologies. The mean age at surgery was 16 years (minimum 13, maximum 19 years). The mean follow-up duration was 6 years (minimum 2, maximum 9 years) without any revision. Regarding the Harris and Oxford hip scores, the mean score were 81 and 39.5 for clinical and patient-related outcomes respectively. The Spearman correlation test revealed a statistically significant positive correlation between the 2 scores of ρ = 0.811 with a *P* value < .001.

**Conclusions:**

THA in adolescents and young adults suffering from end-stage osteoarthritis due to pediatric hip disorders provides improved hip function and notable pain relief at short-term to midterm follow-up.

## Introduction

Total hip arthroplasty (THA) is one of the most performed surgeries in orthopaedics today with good results regarding functional outcomes and pain relief [1,2]. Primary osteoarthritis of the hip is the main etiology for the need of a THA with a mean age of 69 years at surgery [3].

For younger patients, etiologies are quite different [4]. Paediatric hip disorders are thought to account for about 9%-10% of all primary THAs [2].

Perthes disease, posttraumatic arthritis, juvenile inflammatory arthritis (JIA), avascular necrosis (AVN) of the femoral head secondary to slipped capital femoral epiphysis (SCFE), developmental dysplasia of the hip, and septic arthritis are the most encountered paediatric hip disorders that can lead to THA [[4], [5], [6]]. Despite the advances in hip conservation surgery and an increasingly aggressive surgical approach in recent years, there are still adolescents in whom the hip disease progresses to end-stage osteoarthritis [5,7,8]. THA then seems to be the ultimate solution to improve the quality of life of these young patients [9]. According to past literature, THA in adolescents and young subjects seems to be attributed to poorer results than in elderly subjects with primary hip osteoarthritis [10]. Other concerns are the revision surgeries because of aseptic loosening of components due to the use of early-generation bearings and cemented implants. Indeed, these could not accommodate to the high physical demands of young and active patients [2,11]. Furthermore, due to previous surgeries, muscle wasting, scarring, and retained implants, THA in these patients is often technically demanding. Therefore, orthopaedic surgeons continue to show some reluctance to offer THA in very young patients. Nevertheless, due to modern implants and surgical techniques, implant survivorship has increased, and outcomes for THA in the young patient have improved [10,[12], [13], [14]]. Especially, the introduction of ceramic-on-ceramic components as well as highly cross-linked polyethylene has shown reduced wear compared to prior polyethylene components, making THA a more attractive treatment option for young patients with end-stage hip pathology [15,16]. Recent studies show excellent results of THA combined with contemporary prosthetic designs in young patients [10,13]. In addition, beyond the importance of survivorship of THA in young patients, functional outcome is often even more important, as they want to return to an active lifestyle.

Regardless of the encouraging results of the past years on survival of THA in very young patients as described above, there is a lack of literature about patient-related outcomes in adolescents and very young patients.

This study aims to assess the quality of life by analyzing functional and patient-related outcomes at short-term to midterm follow-up after THA performed before the age of 20 years in adolescent and young adults at our center.

Our hypothesis is that adolescents and young adults have a high degree of satisfaction after placement of a THA because of the often very severe and advanced underlying hip pathologies.

## Material and methods

After obtaining approval from the Local Ethics Review Committee (ID Number 2020-02042) and informed consent of the patients, we performed a search of our institutional database for THAs done at our tertiary referral center between January 2008 and December 2018.

We included all patients that underwent a THA before the age of 20 years with a minimum follow-up of 2 years. Patients presenting an underlying neurodevelopmental disease were excluded.

**Figure 1:**
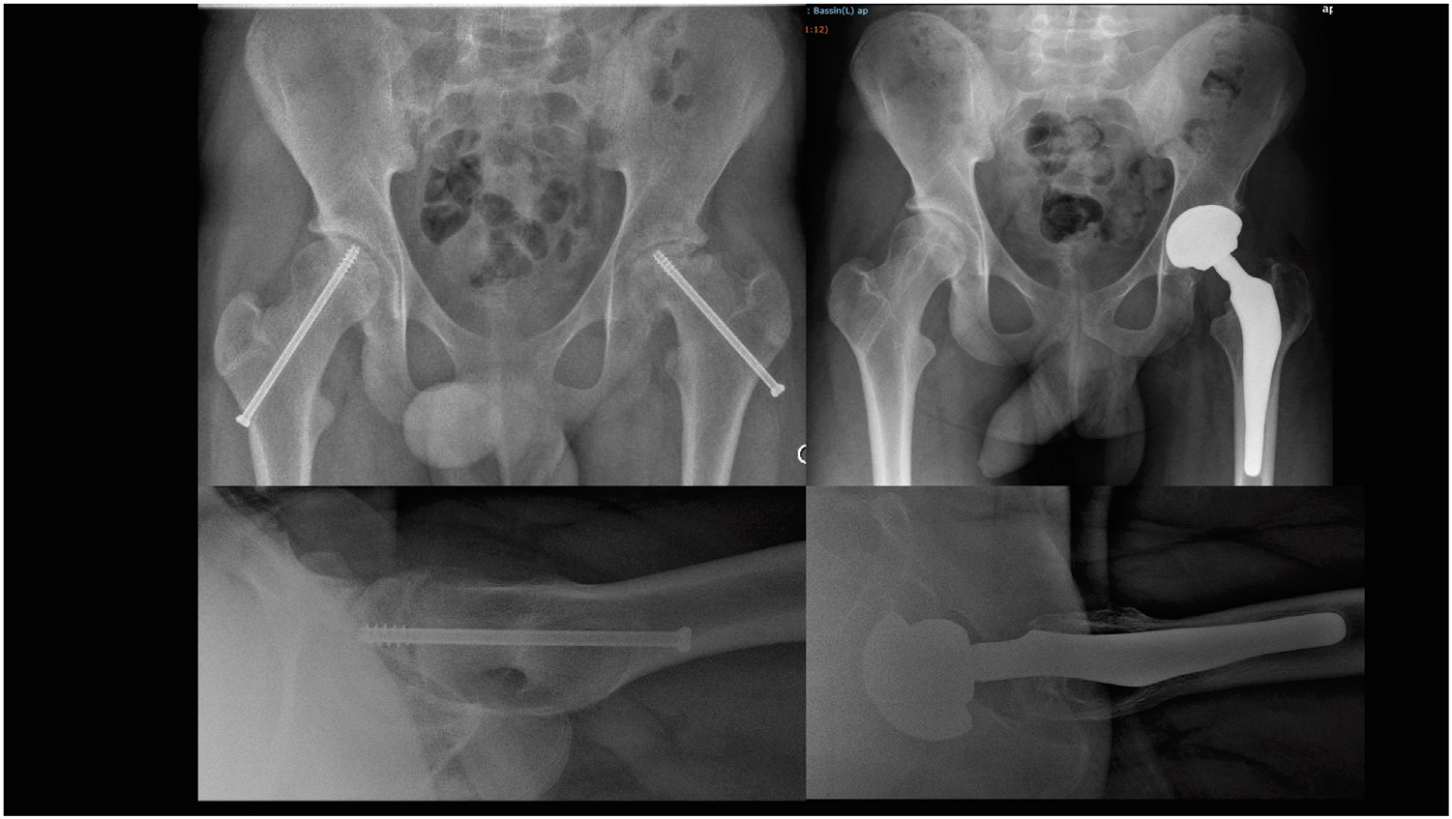
Fifteen-year-old boy with AVN secondary to SCFE who benefited from a customized total hip arthroplasty (Symbios, Switzerland).

All surgeries were performed by the same senior surgeon (P.-Y.Z.). The standard posterior approach was used in every case, with uncemented custom-made femoral and acetabular components and ceramic-on-ceramic bearings (Symbios; Orthopedic society, Switzerland) (Fig. 1).

Computerized three-dimensional preoperative planning was performed in every patient.

Patient-related outcomes have been measured via the Oxford hip score [17]. The Harris hip score was used to assess functional outcomes [18].

We used telephone calls and internet searches to locate patients who were lost to follow-up.

Etiology, previous surgeries, gender, and age at the moment of THA were recorded.

Plain pelvic radiograph and axial hip views were analysed by a senior surgeon (S.R.M.) looking for signs of aseptic loosening.

Complications were recorded and classified according to system discussed in the study by Sink et al. [19].

### Statistical analysis

We performed a descriptive statistical analysis of the demographic data.

Variables were reported as percentages, continuous variables as mean with their corresponding standard deviation (SD), and discrete variables for the Harris hip score and the Oxford hip score were reported as median with their corresponding interquartile range (IQR) given the low number of data and their great variability.

The Spearman correlation test was used to describe the relationship between the Harris and Oxford hip scores. Statistical analyses were done with the R Project for Statistical Computing Version R 4.1.3.

The results were considered significant if the *P*-value was less than or equal to .05.

## Results

After the analysis of our database, we registered 17 patients with a total of 20 THAs.

Of them, 6 patients were lost to follow-up. Finally, 11 patients with a total of 12 THAs were left for final evaluation. One patient presented with bilateral involvement. The left hip was involved in 9 cases (82%). Six patients (54.5%) were male. The mean age at surgery was 16 years (minimum 13, maximum 19). The mean age at the last follow-up was 22 years (minimum 17, maximum 26), with a mean follow-up period of 6 years (minimum 2, maximum 9).

Body mass index at the time of surgery was 22.31 kg/m^2^ (±SD 4.07).

Eight hips (66.6%) had a previous surgery before the THA. All implants were custom-made uncemented THAs with ceramic-on-ceramic pairing.

The following underlying pathologies were encountered: Three patients (4 hips) (27.3%) suffered from JIA; 4 patients presented sequelae of SCFE (36.4%), notably AVN; 2 patients presented posttraumatic AVN of the femoral head (18.2%); 1 patient had sequelae of developmental dysplasia of the hip (9%); and finally, the last patient experienced severe idiopathic chondrolysis of the femoral head (9%).

Analysis of the radiographs at the last follow-up showed no signs of stem subsidence or aseptic loosening.

At the time of the last follow-up, no patient had undergone a revision surgery. Demographics can be seen in Table 1.

**Table 1:** Patients' demographics.

Patient	Hip	Etiology	Age	Gender	Side	Previous surgery	BMI (Kg/m^2^)	Follow-up (y)	Harris hip score	Oxford hip score	Revision surgery
1	1	JIA	18	F	Left	None	24.24	4	92	44	No
2	2	SCFE	15	M	Left	In situ fixation	24.66	7	92	45	No
3	3	Femoral neck fracture	14	F	Left	CRIF	21.5	3	87	41	No
4	4	JIA	17	M	Left	None	n.a	9	100	48	No
5	5	SCFE	15	M	Left	In situ fixation	24.9	2	92	44	No
6	6	Chondrolysis	16	M	Left	None	31.7	3	62	43	No
7	7	SCFE	19	M	Left	Dunn osteotomy	20	6	78	34	No
8	8	SCFE	13	F	Right	In situ fixation	16.2	4	89	46	No
9	9	DDH	13	F	Left	Acetabuloplasty	18.8	8	84	36	No
10	10	Pertrochanteric fracture	19	M	Right	In situ fixation	21.8	6	90	42	No
11	11	JIA	17	F	Right	None	20.78	11	64	32	No
	12				Left	None		10	44	19	
Average			16				22.31	6	81	39.5	No

BMI, body mass index; CRIF, closed reduction and internal fixation; DDH, developmental dysplasia of the hip; n.a., not applicable.

Regarding the Harris hip score the median score was 88 (±IQR 17.5), with a mean of 81 (±SD 16.4). The median score for the Oxford hip score was 42.5 (±IQR 8.75) with a mean of 39.5 (±SD 8.1).

The distribution within the Harris hip score was as follows: 41.67% had an excellent score (over 90), 25% a good score (from 80 to 89), 16.67% had a satisfactory score (70 to 79), and 16.67% had an insufficient score. Scores ranged from 44 to 100.

Within the Oxford hip score, the distribution was as follows: 66.7% had an excellent score (over 41), 25% had a good score (between 31 and 40), and 8.33% had an unsatisfactory score (inferior to 30), with the scores ranging from 19 to 48.

The Spearman correlation test revealed a statistically significant strongly positive correlation between the 2 scores (ρ = 0.811, *P* value < .001).

Complications occurred in 3 patient (27%). According to the classification of Sink et al., all complications were of grade II, and no complication of grade III, IV, or V occurred (Table 2) [19].

**Table 2:** Complications of THA in adolescents and young adults according to Sink et al. [19].

Grade	Original definition according to Sink et al^.^ [19]	Number of events	Complication
I	A complication that requires no treatment and has no clinical relevance; there is no deviation from routine follow-up during the postoperative period; allowed therapeutic regimens include antiemetic, antipyretics, analgesics, diuretics, electrolytes, antibiotics, and physiotherapy	-	Not specifically assessed
II	A deviation from the normal postoperative course (including unplanned clinic visits) that requires outpatient treatment: either pharmacologic or close monitoring as an outpatient	3	Anemia (2), allergy to morphine (1)
III	A complication that is treatable but requires surgical, endoscopic, or radiographic interventions or an unplanned hospital admission	0	
IV	A complication that is life-threatening, requires intensive care unit admission, or is not treatable with potential for permanent disability	0	
V	Death	0	

## Discussion

THAs in adolescents and young adults are often challenging because of underlying pathologies, deformities, and previous surgeries. The most encountered pathologies in our study were AVN secondary to SCFE as well as JIA in 33% of cases, which matches with previous published data [8]. Historically, THA in the very young was mostly performed for JIA with a high revision and loosening rate as high as 40% [20,21]. Those studies mostly report the outcome of THA with first-generation polyethylene implants that are known for more polyethylene wear and secondary implant loosening [[22], [23], [24]]. Fortunately, due to improved surgical techniques, implant material, and design, survivorship of THA in younger patient has strongly improved [15,25].

Ceramic-on-ceramic bearing has been shown to produce less osteolysis with improved wear characteristics and, therefore, lower revision surgery rates [26,27].

Our study shows good to excellent patient-related and functional outcomes at short-term to midterm follow-up for young patients with THA. Indeed, 83.4% showed satisfactory to excellent results for the Harris hips score, and 91.7% showed good to excellent results for the Oxford hip score. Most studies report a significant improvement in functional outcome scores [5,28,29]. Among those, the Harris hip score, used in our study, is typically investigated to assess clinical outcomes [30]. Sixty-seven percent reported good to excellent results, with a mean score of 81/100, which correlates with previous research [31]. We assessed the patient-related outcomes with the Oxford hip score. Indeed, in younger patients with THA, it has been suggested to be more appropriate, and it seems to predict the risk of revision within the subsequent 2 years [17,32,33]. The mean score in our study was 39.5 out of 48.

Furthermore, there was a strong correlation between the Harris hip and Oxford hip scores, which indicates that the patient-related outcome is strongly associated with the functional outcome. This was already confirmed by other authors [34].

Several recent studies evaluating the midterm outcomes of a THA in a population aged 14 to 25 years at the time of surgery showed an improvement in the quality of life and designed THA as a safe therapy that should be considered after failed conservation management in young patients [5,10,28,35,36].

Even if we have to deal with a more active category of patients with a high activity level, thus increasing the mechanical stress on the implants, the survival rate with a mean follow-up period of 6 years was 100% in our study.

Recently, a study of 60 primary THAs performed in 51 patients with a mean age of 16 years showed a survival rate of 97% [8].

Another study, using the National Joint Registry Data Set in England, shows that ceramic-on-ceramic bearings were the most frequently used ones and present the lowest failure rate (2%) with survival estimated at 98% at 5 years [37]. Kim et al. even showed no evidence of osteolysis in their cohort of 93 patients at a minimum follow-up period of 10 years, which is in line with our results [38].

The author recognizes the small sample size as a major limitation to the present analysis.

On the other hand, THA in young patients is rare, especially as surgeons show some reluctance to offer these surgeries to young patients who are often very demanding in everyday activities and, therefore, present higher constraints on the different implants with a greater risk of revision. We could highlight good to excellent patient-related outcomes at short-term to midterm follow-up.

The goal of surgical treatment in these young patients is to provide a long-term solution with pain relief and restoration of function. Our data indicate contemporary THA provides improved hip function and stable implant fixation at short-term to midterm follow-up.

Nevertheless, further studies with longer follow-up of new-generation implants and separate study groups according to each pathology are needed to support our results.

## Conclusions

In adolescents and young adults in whom hip disease progresses to severe joint degeneration, THA provides improved hip function and notable pain relief at short-term to midterm follow-up.

## Conflicts of interest

The authors declare there are no conflicts of interest. The study was approved by our Institutional Review Board (IRB), Lausanne, Canton de Vaud, Switzerland (ID Number 2020-02042).

For full disclosure statements refer to https://doi.org/10.1016/j.artd.2023.101100.

## Authors' contributions

1. All authors made a significant contribution to the work reported, whether that is in the conception; study design; execution; acquisition of data, analysis, and interpretation; or in all these areas.
2. All authors have drafted or written or substantially revised or critically reviewed the article.
3. All authors have agreed on the journal to which the article will be submitted.
4. All authors reviewed and agreed on all versions of the article before submission, during revision, the final version accepted for publication, and any significant changes introduced at the proofing stage.
5. All authors agree to take responsibility and be accountable for the contents of the article.

## Appendix A Supplementary Data

Conflict of Interest Statement for Merckaert

Conflict of Interest Statement for Chapot

Conflict of Interest Statement for Zambelli
