# Supplementary material for: Functional and Patient-related Outcomes of Total Hip Arthroplasty in Patients Younger Than 20 Years
Source: Arthroplast Today. 2023 Mar 6;20:101100. doi: 10.1016/j.artd.2023.101100 (PMC10009676; doi:10.1016/j.artd.2023.101100)
Supplement: Conflict of Interest Statement for Zambelli [file mmc3.pdf]

INDIVIDUAL CONFLICT OF INTEREST STATEMENT

American Association of Hip and Knee Surgeons

(Adopted from the American Academy of Orthopaedic Surgeons disclosure statement)

The following form **must be filled out completely and submitted by each author (example, 6 authors, 6 forms).**
All items require a response. If there is no relevant disclosure for a given item, enter "*None.*"

Manuscript Title

Functional and Patient-related outcomes of total hip arthroplasty in patients younger than 20 years

Authors:

Antoine Chapot[1], Pierre-Yves Zambelli[1][2], MD; Sophie Rosa Margaretha[1], MD

1) Centre Hospitalier Universitaire Vaudois, Service de chirurgie orthopédique pédiatrique, Lausanne, Switzerland
2) Centre Hospitalier Universitaire Vaudois, Service d'Orthopédie adulte, Lausanne, Switzerland

---

1. Royalties from a company or supplier (The following conflicts were disclosed)

   NONE

2. Speakers bureau/paid presentations for a company or supplier (The following conflicts were disclosed)

   NONE

3A. Paid employee for a company or supplier (The following conflicts were disclosed)

   NONE

3B. Paid consultant for a company or supplier (The following conflicts were disclosed)

   NONE

3C. Unpaid consultants for a company or supplier (The following conflicts were disclosed)

   NONE

4. Stock or stock options in a company or supplier (The following conflicts were disclosed)

   NONE

5. Research support from a company or supplier as a Principal Investigator (The following conflicts were disclosed)

   NONE

6. Other financial or material support from a company or supplier (The following conflicts were disclosed)

NONE

7. Royalties, financial or material support from publishers (The following conflicts were disclosed)

   NONE

8. Medical/Orthopaedic publications editorial/governing board (The following conflicts were disclosed)

   NONE

9. Board member/committee appointments for a society (The following conflicts were disclosed)

   NONE

Each author must sign AND print or type his/her name, date and submit a separate form

In addition, one BLINDED Conflict of Interest form (no author names used) should be submitted per manuscript with all author disclosures.

Zambelli Pierre-Yves 24.06.2022

Author Name (Print or Type) Author Signature Date
